# Supplementary figures and images for: Alkaloid and acetogenin-rich fraction from Annona crassiflora fruit peel inhibits proliferation and migration of human liver cancer HepG2 cells
Source: PLoS One. 2021 Jul 8;16(7):e0250394. doi: 10.1371/journal.pone.0250394 (PMC8266062; doi:10.1371/journal.pone.0250394)

**A**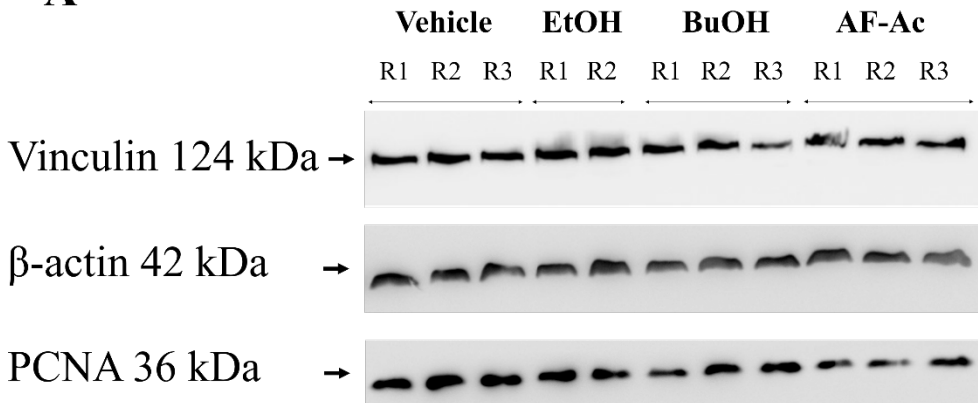**B**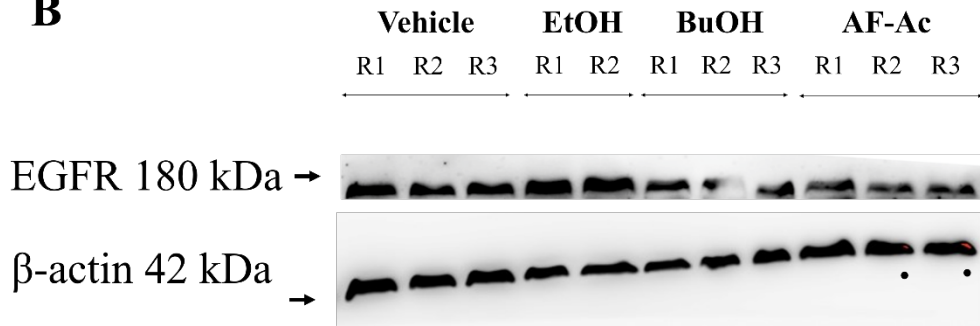

Supplement: S1 Raw images — Six samples of each treatment group were evenly distributed in two gels resulting in 3 samples/gel/group. It was necessary to crop the membranes in order to incubate them with different antibodies since different proteins were analyzed in each western blot. Vinculin (124 kDa MW), β-actin (42 kDa MW) and PCNA (36 kDa MW) were analyzed in the same western blot by cutting the membrane in three (A). The top part was used to blot vinculin antibodies, the middle part was used to blot β-actin antibodies and the bottom part for PCNA antibodies. EGFR (180 kDa MW) and β-actin (42 kDa MW) were analyzed in the same western blot by cutting the membrane in two (B). The top part was used to blot EGFR antibodies and the bottom part for β-actin antibodies. The original blots show results of vinculin, β-actin, PCNA and EGFR expression of HepG2 cells treated with vehicle (control, showed in the three first lanes, included in the present study), crude ethanol extract from A. crassiflora fruit peel (EtOH, not included in the present study), n-butanol fraction from A. crassiflora fruit peel (BuOH, not included in the present study) and alkaloid and acetogenin-rich fraction from A. crassiflora fruit peel (AF-Ac, showed in the last three lanes, included in the present study). The corresponding MW (KD) markers are shown to the left of the Western blot image. (PDF) [file pone.0250394.s001.pdf]

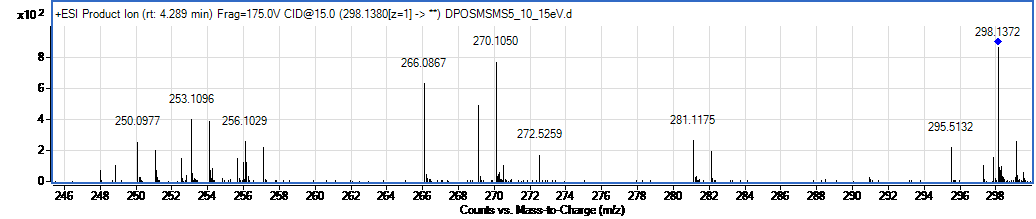

Supplement: S1 Fig — (TIF) [file pone.0250394.s002.tif]

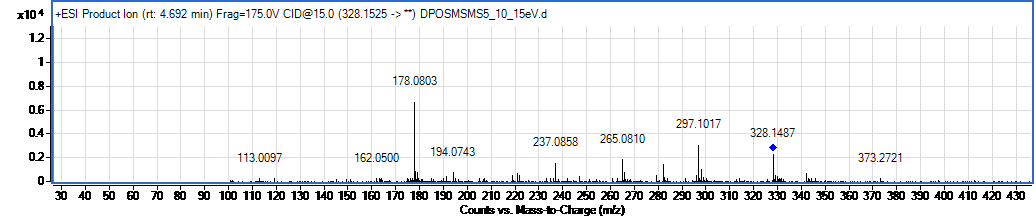

Supplement: S2 Fig — (TIF) [file pone.0250394.s003.tif]

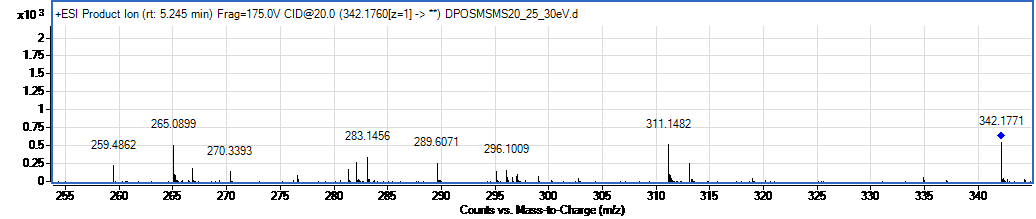

Supplement: S3 Fig — (TIF) [file pone.0250394.s004.tif]

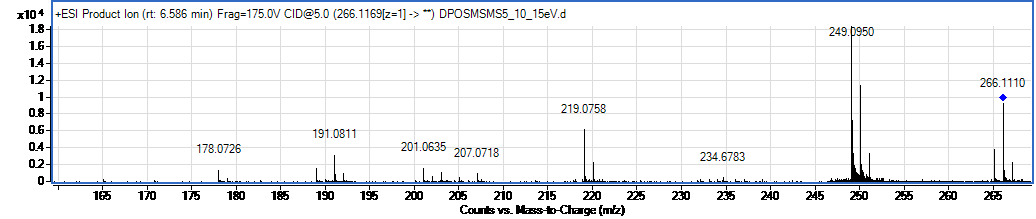

Supplement: S4 Fig — (TIF) [file pone.0250394.s005.tif]

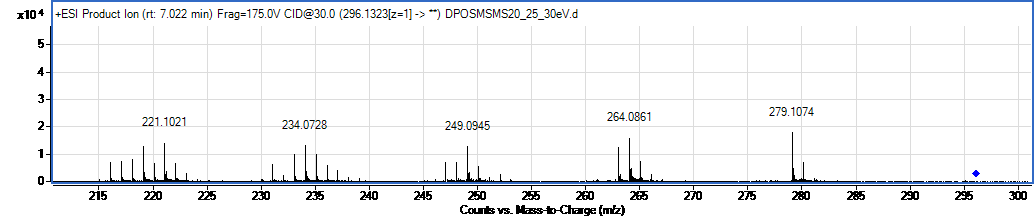

Supplement: S5 Fig — (TIF) [file pone.0250394.s006.tif]

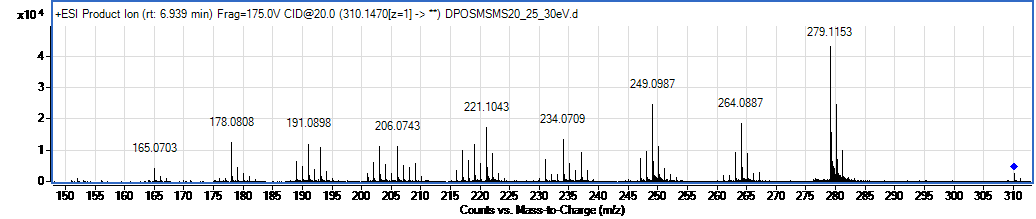

Supplement: S6 Fig — (TIF) [file pone.0250394.s007.tif]

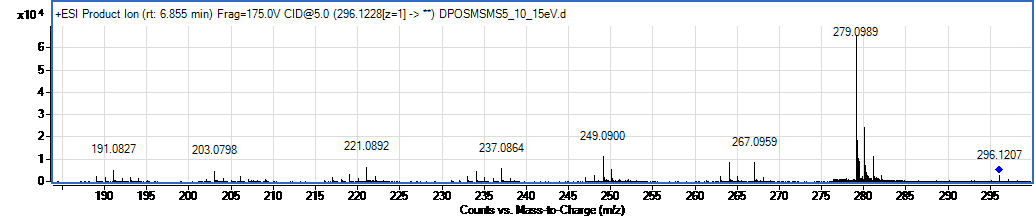

Supplement: S7 Fig — (TIF) [file pone.0250394.s008.tif]

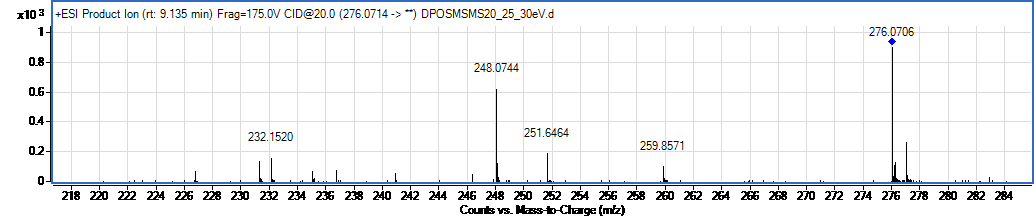

Supplement: S8 Fig — (TIF) [file pone.0250394.s009.tif]

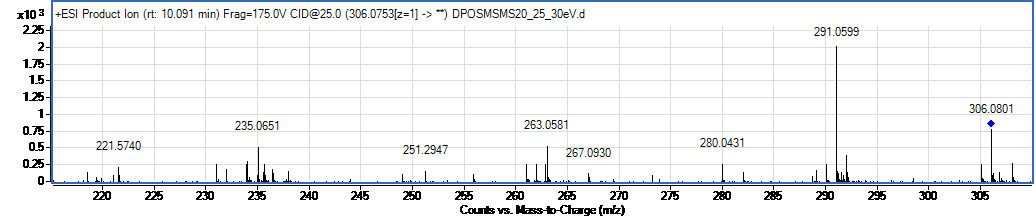

Supplement: S9 Fig — (TIF) [file pone.0250394.s010.tif]

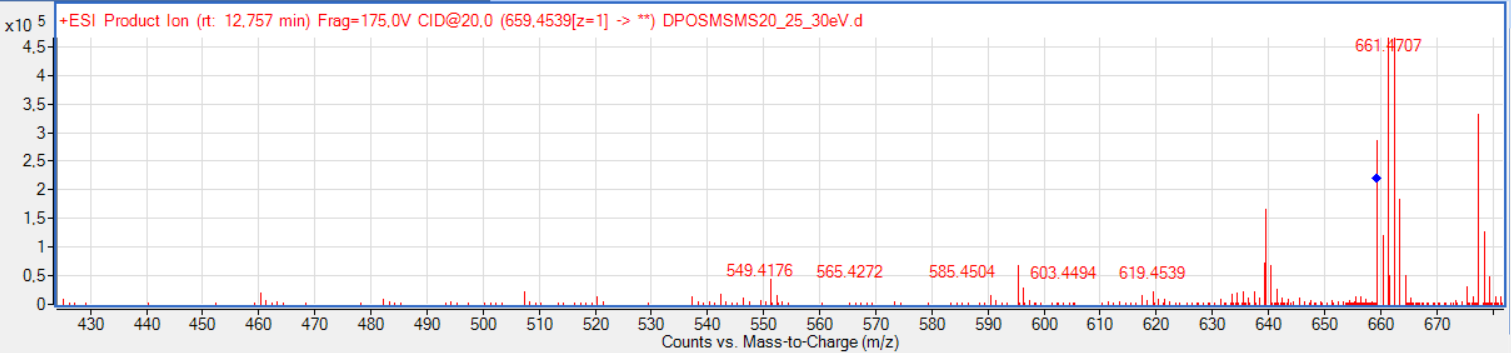

Supplement: S10 Fig — (TIF) [file pone.0250394.s011.tif]

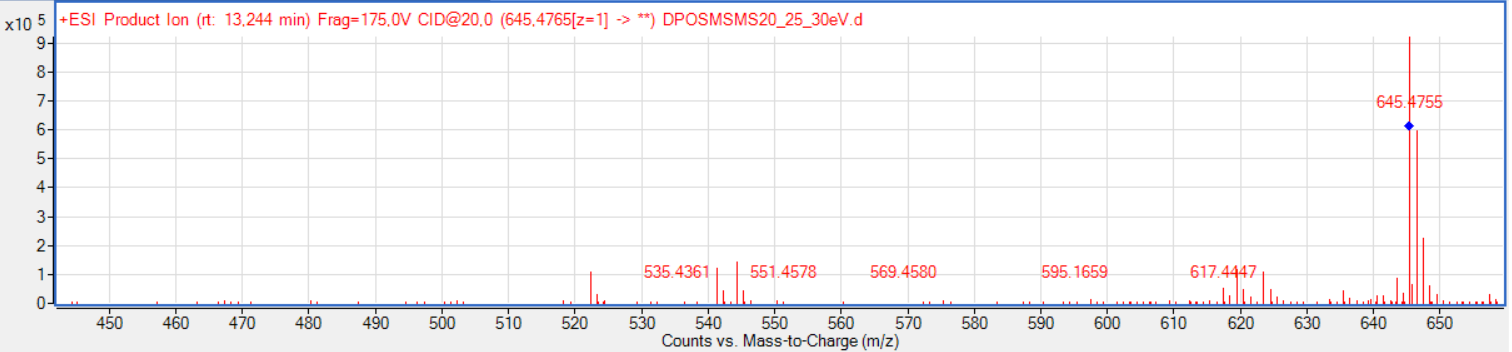

Supplement: S11 Fig — (TIF) [file pone.0250394.s012.tif]

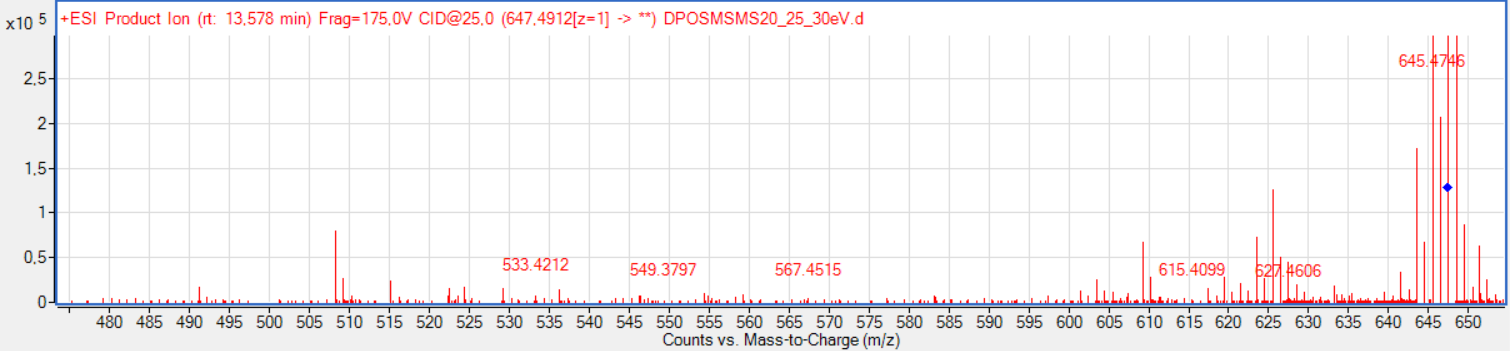

Supplement: S12 Fig — (TIF) [file pone.0250394.s013.tif]

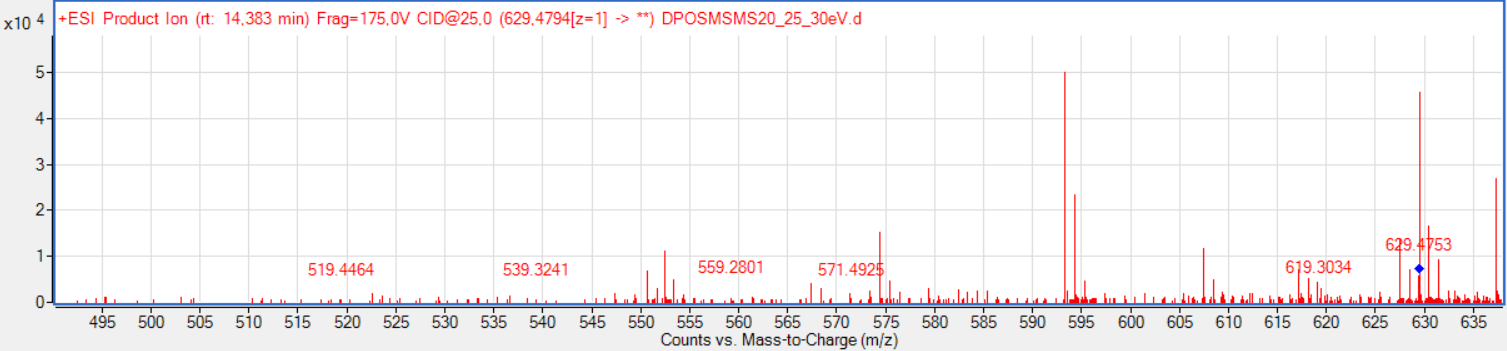

Supplement: S13 Fig — (TIF) [file pone.0250394.s014.tif]

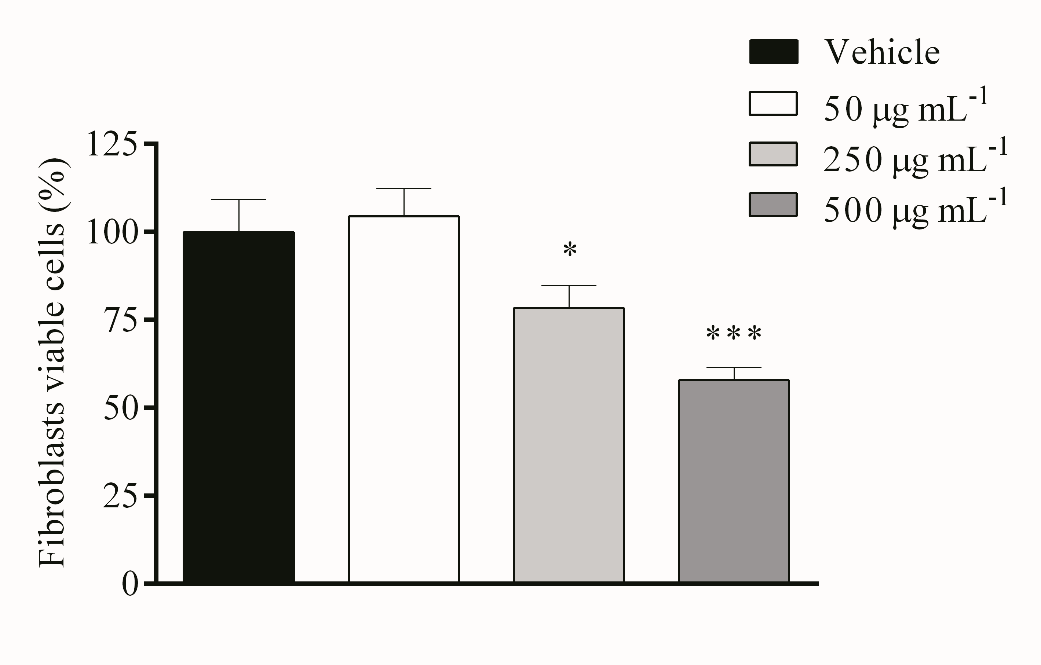

Supplement: S14 Fig — Results (mean ± SD, n = 3) expressed as the percentage of viable cells compared to the vehicle group. Significance levels are indicated by *p < 0.05 and ***p < 0.001 when compared to control (one-way ANOVA and Dunnett as posttest). The fibroblast cells (NIH/3T3) were grown in RPMI-1640 medium supplemented with fetal bovine serum 10% (FBS), 2 mM glutamine, 100 U/mL penicillin and 100 mg/mL streptomycin, at 37°C and CO2 5%. 3 x 10^4^ cells were plated in 96-well plates and treated with different concentrations of the AF-Ac fraction or vehicle and incubated for 24 h at 37°C and 5% CO2. Then, 100 μL of 5 mg/mL (3-(4,5-dimethylthiazolyl-2)−2,5-diphenyltetrazolium bromide) solution was incubated with the supernatant at 37°C for 2 h in 5% CO2. Next, dimethyl sulfoxide (DMSO) was added and the cell viability was analyzed by absorbance of the purple formazan from viable cells at 570 nm (Molecular Devices, Menlo Park, CA, USA). (TIF) [file pone.0250394.s015.tif]

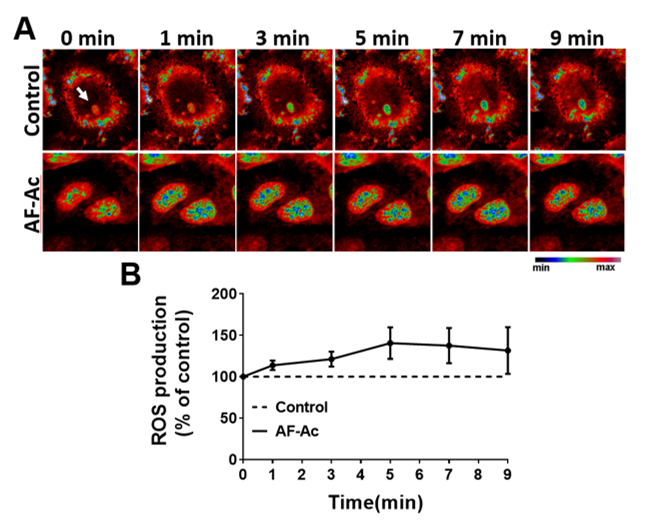

Supplement: S15 Fig — Time-lapse of HepG2 cells labeled with dihydroethidium (DHE) and exposed to the AF-Ac fraction (A). Quantification of the ROS production during 9 minutes after AF-Ac treatment (B). The values are expressed in the percentage of the control. HepG2 cells were labeled with 5 μM DHE. The cells were placed at the confocal stage and perfused with HEPES solution as control and HEPES + AF-AC fraction for 10 min. The fluorescence intensity was measured using Image J and the values are expressed in percentage of the control. (TIF) [file pone.0250394.s016.tif]
